# Using group testing in a two-phase epidemiologic design to identify the effects of a large number of antibody reactions on disease risk

**DOI:** 10.1186/s12874-022-01798-0

**Published:** 2022-12-16

**Authors:** Tanvi Mehta, Yaakov Malinovsky, Christian C. Abnet, Paul S. Albert

**Affiliations:** 1 Division of Cancer Epidemiology and Genetics, National Cancer Institute, 9609 Medical Center Drive, Room SG/7E146, Rockville, MD 20850 USA; 2 Department of Mathematics and Statistics, University of Maryland, Baltimore County, Baltimore, MD USA

**Keywords:** Case-control studies, Epidemiologic design, Group Testing, Prevalence estimation

## Abstract

**Background:**

The role of immunological responses to exposed bacteria on disease incidence is increasingly under investigation. With many bacterial species, and many potential antibody reactions to a particular species, the large number of assays required for this type of discovery can make it prohibitively expensive. We propose a two-phase group testing design to more efficiently screen numerous antibody effects in a case-control setting.

**Methods:**

Phase 1 uses group testing to select antibodies that are differentially expressed between cases and controls. The selected antibodies go on to Phase 2 individual testing.

**Results:**

We evaluate the two-phase group testing design through simulations and example data and find that it substantially reduces the number of assays required relative to standard case-control and group testing designs, while maintaining similar statistical properties.

**Conclusion:**

The proposed two-phase group testing design can dramatically reduce the number of assays required, while providing comparable results to a case-control design.

**Supplementary Information:**

The online version contains supplementary material available at 10.1186/s12874-022-01798-0.

## Background

Group testing procedures have been used for disease screening and prevalence estimation since the early 1940s [1]. With group testing, rather than separately testing individual samples for a binary biological response, samples are pooled together into a group and a group assessment of positivity is determined. Two major uses of group testing are in disease status identification and prevalence estimation. For disease identification, the goal is to test samples in groups with the purpose of fully identifying all disease cases with the fewest numbers of tests [2 and references within]. A common strategy is to test a group that consists of combined samples and to only continue further if the group outcome is positive; otherwise, one would stop and conclude all samples in the group are disease negative. On the other hand, we only need the group outcomes (without necessarily individual identification) for prevalence estimation [3 and references within for a literature review]. Group testing designs have increasingly been used as a cost-effective alternative to individual testing in the biosciences [4]. This paper proposes a novel two-phase group testing design for identifying case-control differences among many antibodies in an epidemiologic setting.

In the first phase of the proposed design, the prevalence of antibody reactivity is estimated in cases and controls using only the combined sample results from group testing without individual retesting. Zhang et al. and references within investigate situations where retesting positive pools results in efficiency gains for prevalence estimation [5]. In a similar vein, we retest positive pooled samples with individual tests, but to reduce the number of tests, we do this only for antibodies with preliminary statistical evidence for a case-control difference. This new design is compared to a case-control design with individual testing on all antibodies and to a standard group testing design where positive pooled samples are retested for all antibodies without regard to examining preliminary case-control differences.

The role of immunological responses to exposed bacteria on disease incidence is increasingly under investigation. New technologies for identifying many antibody-specific reactions to particular bacterial exposures are being developed and used in epidemiologic settings [6]. With many (> 1,000) potential antibody reactions to a bacterial species, and multiple (> 15) potential species being examined in a single study, this analysis may be high dimensional (*n* > 15,000), and therefore may be prohibitively expensive.

This two-phase group testing design is motivated by a recent study focused on screening for case-control differences in *Helicobacter pylori* antibodies to better understand risk of gastric cancer [6]. Since this was the first study of this type, it focused on only one bacterial species (*Helicobacter pylori*), but with additional species, future studies may analyze over 15,000 antibodies. Our aim is to develop a design to minimize the number of serologic tests required in this type of setting. We propose a two-phased approach for the efficient detection of antibody case-control differences (with the goal of identifying potential target antibodies for further investigation) where group testing is used in the first phase to select a subset of antibodies with preliminary evidence for a case-control difference and individual samples are retested on positive pooled samples within the subset during the second phase. We show how to implement this approach, and through simulations, demonstrate the substantial reduction in the number of serologic tests required relative to a standard case-control design.

## Methods

**Fig. 1:**
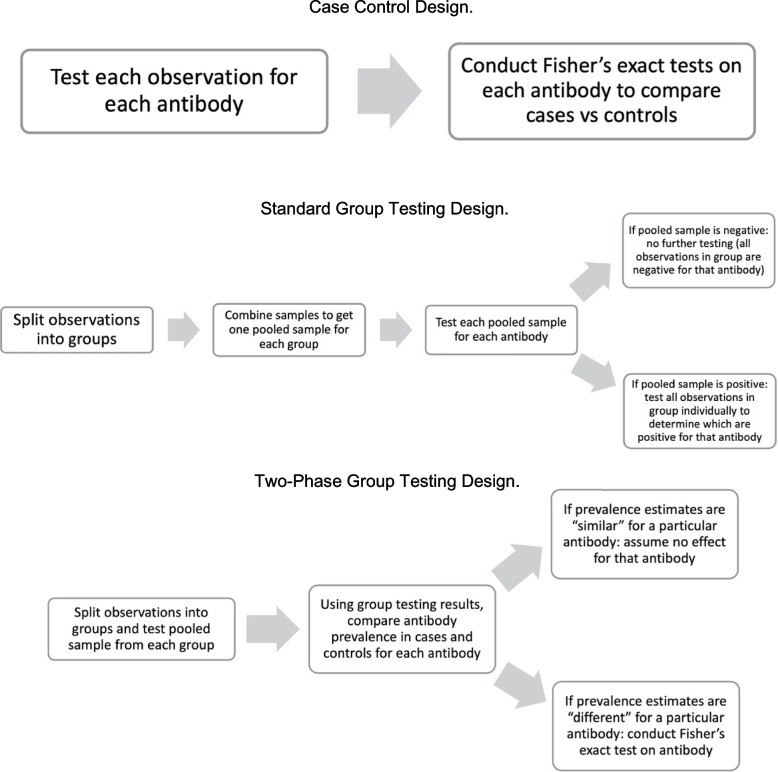
Diagrams depicting the three different designs that are compared in this paper

An analysis of the case-control study without group testing would require a direct comparison of the frequency of antibody-specific reactions between cases and controls across the large number of antibodies, as depicted in Fig. 1. These frequencies are usually based on thresholding a quantitative serological assay or can be directly assessed with a qualitative assay that is inherently dichotomous. The case-control analysis with 15,000 antibodies would require researchers to analyze 15,000 multiplied by the total study sample size in number of assays. The large number of assays required for a sufficiently powered study would make this approach infeasible. We propose a group testing strategy to substantially reduce the number of required assays (tests) without sacrificing much power.

Our inferential goal is to test for case-control differences for each antibody where we control the point-wise error rate (e.g., each antibody-specific comparison between cases and controls has a type I error rate of $\alpha$). We recognize the number of antibodies is large and that we would expect an average number of false discoveries of $\alpha$ multiplied by the number of antibodies.

We propose a two-phase design where in the first phase we screen antibodies using group testing and only proceed to a second stage when there is a good indication of an effect. We describe the procedure as follows.

### Phase 1

In phase 1, we use group testing to estimate the prevalence of antibodies, compare the prevalence estimates between cases and controls, and use this comparison to select antibodies. We split the observations by case-control status into groups of equal size. We then test each group for each antibody. We estimate the case and control prevalences for each antibody using the Burrows estimator [7, 8] and references within. This estimator is given by

$$\widehat p=1-\left(1-\frac x{n+v}\right)^\frac1k\;with\;v=\frac{k-1}{2k}$$

where *x* is the number of positive groups, *k* is the group size, and *n* is the number of groups.

Although prevalence can be estimated using maximum-likelihood (MLE), this estimator will be biased. An alternative estimator was proposed by Burrows that eliminates most of the bias. In addition, Burrows showed empirically that his estimator not only improves on the bias but yields a smaller mean-square error (MSE) than the MLE for all values of p considered (*p* ≤ 0.5) [7].

We compute a two-sided z-test for each antibody to evaluate evidence for a case-control difference,

$${Z}_{antibody}=\frac{{\widehat{p}}_{case}-{\widehat{p}}_{control}}{\sqrt{\widehat{var}\left({\widehat{p}}_{case}\right)+\widehat{var}\left({\widehat{p}}_{control}\right)}}$$

where ${\widehat{p}}_{case}$ and ${\widehat{p}}_{control}$ are Burrow’s estimators for the cases and controls, respectively. The variances of these estimators are computed as

$$\widehat{var}\left(\widehat p\right)=\frac{\left(1-\theta\right)\left(1-\widehat p\right)^2}{k^2}\ast\left(\frac1{n\theta}+\frac{2\left(1-\theta\right)v^2}{(n{\theta)}^2}\right)-\left(\frac{v\left(1-2v\right)\left(1-\theta\right)\left(1-\widehat p\right)\left(1+\theta\right)\left(1-v\right)}{6n^2\theta^2}\right)^2with\;\theta:\left(1-\widehat p\right)^\text{k}$$

We then use the two-sided *p*-value from the calculated z-statistic to determine whether there is enough evidence of a difference for that antibody to advance to phase 2 individual testing. If the *p*-value is less than the phase 1 cutoff (c_1_), we conduct individual testing; if it is greater, we assume there is no effect.

### Phase 2

For those antibodies that proceed to Phase 2, we conduct a Fisher’s exact test and conclude there is a case-control difference if the resulting *p*-value is less than cutoff c_2_. The type I error rate of the final test is a function of both c_1_ and c_2_. Therefore, given c_1_, we need to determine c_2_ to control the final type I error rate at the nominal $\alpha$ level.

### Calibration of c_2_

We use a Monte-Carlo approach to compute c_2_ as a function of the antibody prevalence, by applying the two-phase design to data that was generated under the null hypothesis of no case-control effects, with 10,000 realizations for each prevalence value.

**Fig. 2:**
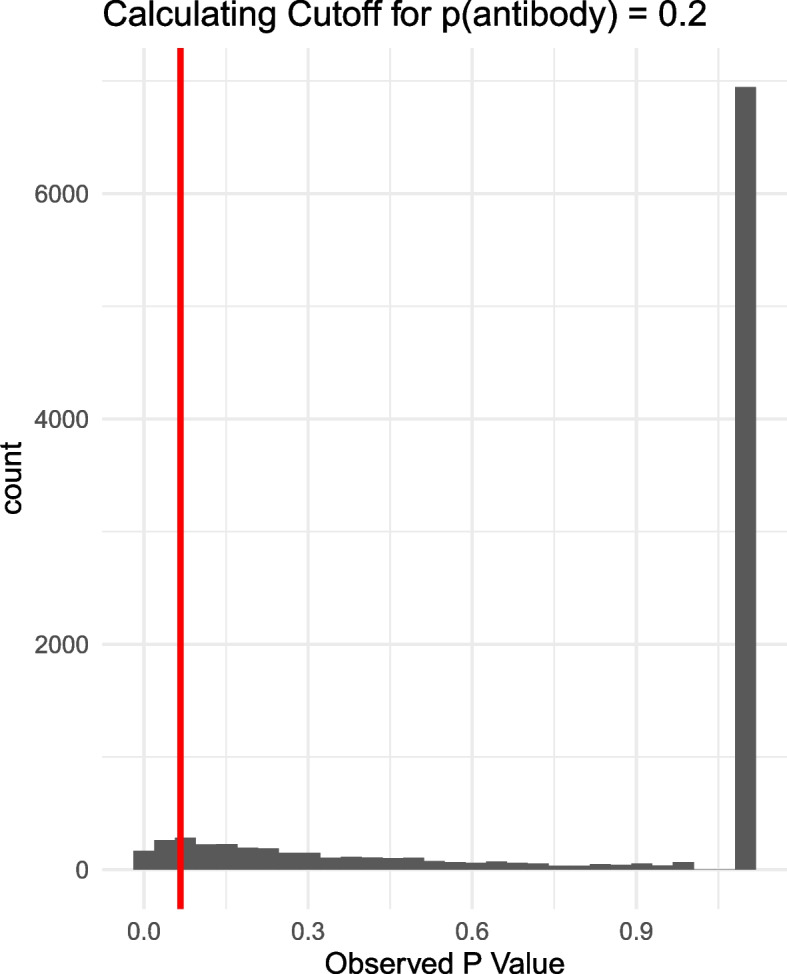
Plot of the null distribution of 10,000 Fisher’s exact test *p*-values under the two-phase design with group size 5 from Monte Carlo approach for an antibody prevalence of 0.2. Large column on the right shows those instances that did not advance to phase 2, and therefore are considered not significant. Red vertical line indicates the 5th percentile of all *p*-values, and therefore the determined phase 2 cutoff that should be used to maintain an overall rejection rate and alpha level of 0.05

**Fig. 3:**
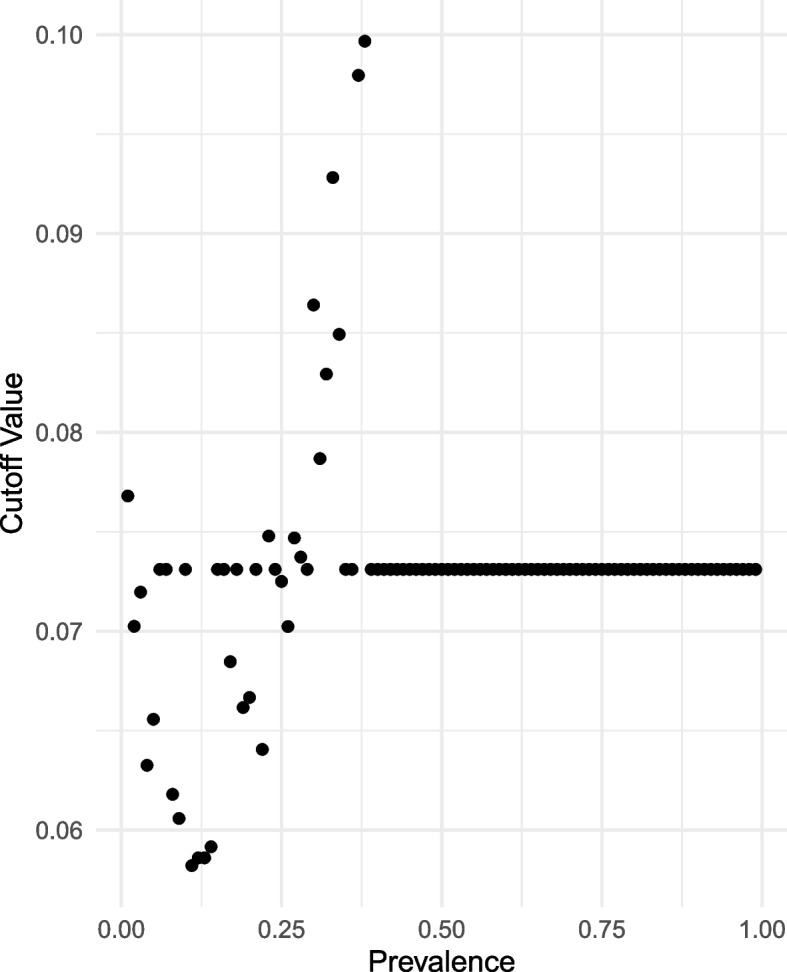
Plot of the Monte-Carlo *p*-value cutoffs for each antibody prevalence between 0 and 1 with step size 0.01. For high prevalences (> 0.4) and any prevalences with too few instances advancing to phase 2, the mean of the remaining cutoffs was used

Figure 2 illustrates how to choose the *p*-value used in phase 2 testing to achieve a final $\alpha$ level test. The figure shows the observed *p*-values in phase 2 testing under the null distribution, for an example antibody prevalence of 0.20. The $1-\alpha$ percentile of the resulting phase 2 *p*-values determines the cutoff value c_2_. Rather than applying the Monte-Carlo procedure for each of the large number of antibodies (e.g., 15,000), we evaluate c_2_ as a function of prevalence by partitioning prevalence in units ranging from 0 to 1 by steps of size 0.01 (this requires only performing 100 Monte-Carlo simulations). Figure 3 shows the Monte-Carlo *p*-value cutoffs (c_2_) as a function of prevalence, and these values were used for phase 2 testing. Noting that the resulting curve was not continuous, we also applied Lowess smoothing in order to construct a continuous curve of c_2_ as a function of prevalence. However, smoothing the curve showed little differences in testing characteristics relative to simply interpolating between discrete sequence values so we used the non-continuous values for simplicity. We identified statistically significant antibody effects by comparing the *p*-value from the Fisher’s exact test to c_2_.

#### Standard group testing approach

An alternative to the two-phase design described above uses group testing to reconstruct the complete data. In this design, group testing is applied to the entire dataset in the following manner: for groups that are negative, we assume all individuals in that group are negative and for groups that are positive, we retest individual samples to reconstruct the individual data on which standing Fisher’s exact tests can be applied.

### Simulation

We compare the proposed two-phase design with both a standard case-control and group testing design in terms of expected numbers of tests and statistical power. We generate data of 15,000 antibodies for 500 cases and 500 controls. The probability for a particular antibody *j* for individual *i* is given by

$${p}_{antibod{y}_{ij}}={\Phi }({\alpha }_{0j}+{\alpha }_{1j}{y}_{i}+{b}_{i})$$

$$\mathrm{with}\;{\mathrm\alpha}_{0\mathrm j}\overset{}{\sim\mathrm N}(-3,0.5)\;;\;\alpha_{1[1-200]}=0.73\;and\;\alpha_{1[201-\text{15,000}]}=0;$$

$$y_i=\left\{\begin{array}{cc}1&case\\0&control\end{array}\right.;b_i\overset{}{\sim N}(0,2)\;;\;\Phi\;\text{is the cumulative distribution function of the N}(0,1)\;\text{distribution}$$

With the resulting antibody probabilities ($mean\left({p}_{antibod{y}_{ij}}\right)=0.1$), we generate 15,000 antibody outcomes for each individual using a binomial distribution. The random effect ${b}_{i}$ incorporates an exchangeable correlation structure between antibody responses on the same individual.

The 0.73 in the above equation reflects the case-control differences on the probit scale for the first 200 antibodies. The remaining 14,800 antibodies have no case-control differences. In the following simulations we evaluate power based on the first 200 antibodies and type I error from the remaining antibodies.

## Results

### Simulation results

The two-phase design requires investigators to specify c_1_. Choosing a value of c_1_ too large (close to 1) results in the progression to phase 2 for a large number of antibodies which will lead to a large number of tests. On the other hand, choosing a value of c_1_ that is too small will result in a small number of tests, but will have low power. As a compromise we chose c_1_ = 0.3; later in the simulation we investigate this choice. We performed 1000 Monte-Carlo repetitions and choose the $\alpha$ level to be 0.05.

The comparison between the different designs is presented in Table 1. There is a large reduction in the expected number of tests with two-phase group testing relative to the case control design. The two-phase design with group size 5 has similar statistical properties (power and type I error rate) to the case-control design and uses only 32% of the tests. The standard group testing design with a group size of 5 has the same statistical properties as the case-control design while still reducing the number of tests but uses 58% of the tests used in the case-control design. For a larger group size (group size of 10), the two-phase design performs well (similar to a group size of 5), while the standard group testing design is less efficient.

**Table 1:** A comparison of the power, type 1 error, and expected or actual number of tests used for all three designs. Group sizes of 2, 5, and 10 were used in the group testing designs

		Power	Type I Error	(Expected) # of Tests
Case Control Design		0.843	0.041	15,000,000
Standard Group Testing Design	Group Size 2	0.843	0.041	10,163,667
	Group Size 5	0.843	0.041	8,713,995
	Group Size 10	0.843	0.041	10,498,037
Two-Phase Group Testing Design	Group Size 2	0.867	0.055	8,356,222
	Group Size 5	0.859	0.053	4,775,891
	Group Size 10	0.860	0.060	4,125,408

We examined the sensitivity of the simulation results to the choice of c_1_ at alternative values of 0.1, 0.2, 0.3, and 0.4 in Table 2. All choices resulted in substantial efficiency gain relative to the case-control design. Choosing c_1_ at 0.2 or 0.3 appears to be a good balance between power and the expected numbers of tests.

**Table 2:** Power, false positive rate and expected number of test results for varying values of c_1_ with a consistent group size of 5

Stage 1 Cutoff	0.1	0.2	0.3	0.4
Power	0.829	0.875	0.859	0.907
False Positive Rate	0.048	0.059	0.053	0.067
Expected # of Tests	3,666,336	4,217,337	4,775,891	5,452,877

For the antibody testing conducted in this epidemiologic setting, there is little evidence for dilution error in the range of group sizes we are considering. Particularly, perfect sensitivity is expected. That said, we conducted a simulation study examining the properties of the proposed group testing method under losses of sensitivity. Table 3 shows the operating characteristics for a c_1_ cutoff of 0.3 for a sensitivity of 0.95 and 0.98. The results are nearly indistinguishable from the case of perfect sensitivity shown in Table 2.

**Table 3:** Power, false positive rate and expected number of test results for varying levels of dilution error (losses of sensitivity) with a consistent group size of 5

Sensitivity	0.950.98	0.98
Power	0.877	0.886
False Positive Rate	0.058	0.062
Expected # of Tests	4,705,193	4,747,283

### Example results

We analyzed the case-control study data described in the introduction (3,055 antibodies in 50 cases and 50 controls with group size 5) [6] using the case-control, standard group testing, and two-phase group testing designs. Antibody serology was normalized relative to the median raw expression values for all proteins on a given array and a value of 2 was chosen as the threshold for determining antibody positivity based on the experience of the laboratory [6].

We found that the case-control design identified four antibodies at the 0.05 significance level. The two-phase design identified the same four antibodies. With a small sample size, we would anticipate low power for identifying antibody effects. In practice, studies will have larger sample sizes. We evaluated this by resampling a larger number of cases and controls from the original dataset (resampling with replacement from the original dataset, creating a dataset with 500 cases and 500 controls).

We investigated designs with group sizes of 5, 10, and 20. Results are shown in Table 4. Under the case control design, 642 of 3,055 antibodies are significant and 2,413 are not significant. Of the 642 antibodies that are significant under the case control design, 641 antibodies are significant under the two-phase design for a group size of 5; 635 and 621 are significant for group sizes of 10 and 20, respectively. Of the 2,413 antibodies that are not significant under the case control design, 2,400, 2,399, and 2,401 are not significant with a two-phase design with group sizes of 5, 10, and 20 respectively. Table 5 shows the expected number of tests under a two-phase design for different group sizes. The two-phase design is substantially more efficient with respect to the expected number of tests as compared with the case-control design. The case control design uses 3,055,000 tests, while the two-phase group testing design with group size 10 uses 1,024,400 tests, only 34% of the tests required by the case control design. Since many of the antibody prevalences are small, a group size of 20 was more efficient than designs with a smaller number of groups (5 or 10). However, even with a group size of 5, the number of tests required for the two-phase design is less than half (1,335,000 compared with 3,055,000).

**Table 4:** Concordance of Antibody Identification Among Designs when Applied to Example Data. Results of implementing the designs on resampled example data, comparing the case control design and two-phase group testing design with group sizes 5, 10, and 20. Note that the standard group testing design will identify the same significant antibodies as the case control design, so results are not explicitly listed for simplicity

Case Control Design		Number of Antibodies Significant	
		642	
Two-Phase Group Testing Design		Number Significant of Significant in CC	Number Not Significant of Significant in CC
	Group Size 5	641 *(99.84%)*	1 *(0.16%)*
	Group Size 10	635 *(98.91%)*	7 *(1.09%)*
	Group Size 20	621 *(96.73%)*	21 *(3.27%)*
Case Control Design		Number of Antibodies Not Significant	
		2,413	
Two-Phase Group Testing Design		Number Significant of Not Significant in CC	Number Not Significant of Not Significant in CC
	Group Size 5	13 *(0.54%)*	2,400 *(99.46%)*
	Group Size 10	14 *(0.58%)*	2,399 *(99.42%)*
	Group Size 20	12 *(0.50%)*	2,401 *(99.50%)*

Although the two-phase design is more efficient than the case-control design, Table 5 shows that it is not as efficient as a standard group testing design. The high efficiency of the standard group testing design is due to the high cutoff value of 2, which resulted in an overall low antibody reactivity rate of 0.008. In the Supplement, we compared the case-control, standard group testing, and the proposed two-phase group testing design for antibody reactivity cutoffs of 1.25 and 1.15, corresponding to antibody prevalences of 0.09 and 0.18, respectively. The two-phase group testing design is shown to have improved efficiency relative to the standard group testing design for these larger prevalences.

**Table 5:** Expected Number of Tests by Design with Example Data. The number of tests used for the case control design, the standard group testing design, and the expected number of tests for the two-phase group testing design when these designs were applied to the resampled example data

		Number of Tests
Case Control Design		3,055,000
Standard Group Testing Design	Group Size 5	702,725
	Group Size 10	453,690
	Group Size 20	377,370
Two-Phase Group Testing Design	Group Size 5	1,335,000
	Group Size 10	1,024,500
	Group Size 20	849,750

## Discussion

Through simulations and example data, we see that the proposed two-phase group testing design as compared with either case-control or standard group testing designs can dramatically reduce the number of tests required while maintaining similar power. In the applied setting we are considering, the prevalence is generally low. When antibody prevalence is high, group testing approaches will not be as efficient as a case-control comparison. With higher antibody prevalence, an alternative design introducing an intermediate phase (e.g., collect a small sample of individual data if the phase 1 case and control prevalences are larger than 0.4) may be useful. Such a design requires further exploration.

The cutoff for positivity is often very difficult to determine for bacterial pathogens (e.g., antibodies such as StrepA where carriage is common). For streptococcal serology an 80th percentile in healthy controls is often chosen as the clinical cutoff for reactivity where 20% of controls would be expected to test positive for an antigen [9]. In such a case, we expect the proposed two-phase group testing approach would show efficiency gains over the case-control and standard group testing designs. In the *Helicobacter pylori* antibodies in the gastric cancer example, the chosen threshold of 2 resulted in an average antibody reactivity rate of < 1%. In this case, the illustrative example suggested that the standard group testing design outperforms both the case-control and two-phase group testing designs. The two-phase design shows efficiency advantages for a prevalence of 9% and 18% (threshold of 1.15 and 1.25, respectively).

We assume there is no dilution error (error because the samples are pooled). Since laboratory procedures for this type of antibody testing require substantially diluting the sample to detect signal, a loss in sensitivity by pooling is not expected for moderate sized groups. However, the pooling could result in a loss of specificity. Through simulations, we demonstrated that the design properties are essentially the same with small losses in specificity in pooled samples. In practice we suggest that researchers conduct validation studies to assure that assuming no dilution is reasonable.

In our application, we are interested in screening antibodies for further investigation, so no adjustments were made for confounding. For other epidemiology applications, it may be important to adjust for covariate effects. The current approach can incorporate discrete covariate combinations by performing the two-phase design stratified by combination group. An extension of the two-phase design to incorporate continuous covariates is less straightforward and is a topic for future research.

## Supplementary Information

**Additional file 1.**

## Data Availability

The data has been made available at github.com/mehtatanvi/Two-Phase-GT-Design.
