# Supplementary material for: Using group testing in a two-phase epidemiologic design to identify the effects of a large number of antibody reactions on disease risk
Source: BMC Med Res Methodol. 2022 Dec 16;22:324. doi: 10.1186/s12874-022-01798-0 (PMC9756457; doi:10.1186/s12874-022-01798-0)
Supplement: Supplementary file 1 — Additional file 1. [file 12874_2022_1798_MOESM1_ESM.docx]

**Supplement**

| Case Control Design |  | Number of Antibodies Significant | |
| --- | --- | --- | --- |
|  |  | 1,701 | |
| Two-Phase Group Testing Design |  | Number Significant of Significant in CC | Number Not Significant of Significant in CC |
|  | Group Size 5 | 1,654 *(97.24%)* | 47 *(2.76%)* |
|  | Group Size 10 | 1,537 *(90.36%)* | 164 *(9.64%)* |
|  | Group Size 20 | 1,275 *(74.96%)* | 426 *(25.04%)* |
|  | | | |
| Case Control Design |  | Number of Antibodies Not Significant | |
|  |  | 1,354 | |
| Two-Phase Group Testing Design |  | Number Significant of Not Significant in CC | Number Not Significant of Not Significant in CC |
|  | Group Size 5 | 64 *(4.73%)* | 1,290 *(95.27%)* |
|  | Group Size 10 | 56 *(4.14%)* | 1,298 *(95.86%)* |
|  | Group Size 20 | 35 *(2.58%)* | 1,319 *(97.42%)* |

Table S1. Concordance of Antibody Identification Among Designs when Applied to Example Data with a Cutoff of 1.25.

Results of implementing the designs on resampled example data with a cutoff of 1.25, resulting in an antibody prevalence of 0.09, comparing the case control design and two-phase group testing design with group sizes 5, 10, and 20.

|  |  | Number of Tests |
| --- | --- | --- |
| Case Control Design |  | 3,055,000 |
| Standard Group Testing Design |  |  |
|  | Group Size 5 | 1,590,415 |
|  | Group Size 10 | 1,726,490 |
|  | Group Size 20 | 1,980,0 |
| Two-Phase Group Testing Design |  |  |
|  | Group Size 5 | 2,747,000 |
|  | Group Size 10 | 2,284,500 |
|  | Group Size 20 | 1,718,750 |

Table S2. Expected Number of Tests by Design with Example Data for Cutoff of 1.25.

The number of tests used for the case control design, the standard group testing design, and the expected number of tests for the two-phase group testing design with the cutoff adjusted to 1.25

| Case Control Design |  | Number of Antibodies Significant | |
| --- | --- | --- | --- |
|  |  | 1,657 | |
| Two-Phase Group Testing Design |  | Number Significant of Significant in CC | Number Not Significant of Significant in CC |
|  | Group Size 5 | 1,521 *(91.79%)* | 136 *(8.21%)* |
|  | Group Size 10 | 1,176 *(70.97%)* | 481 *(29.03%)* |
|  | Group Size 20 | 766 *(46.23%)* | 891 *(53.77%)* |
|  | | | |
| Case Control Design |  | Number of Antibodies Not Significant | |
|  |  | 1,398 | |
| Two-Phase Group Testing Design |  | Number Significant of Not Significant in CC | Number Not Significant of Not Significant in CC |
|  | Group Size 5 | 69 *(4.94%)* | 1,329 *(95.06%)* |
|  | Group Size 10 | 40 *(2.86%)* | 1,358 *(97.14%)* |
|  | Group Size 20 | 20 *(1.43%)* | 1,378 *(98.57%)* |

Table S3. Concordance of Antibody Identification Among Designs when Applied to Example Data with a Cutoff of 1.15.

Results of implementing the designs on resampled example data with a cutoff of 1.15, resulting in an antibody prevalence of 0.18, comparing the case control design and two-phase group testing design with group sizes 5, 10, and 20.

|  |  | Number of Tests |
| --- | --- | --- |
| Case Control Design |  | 3,055,000 |
| Standard Group Testing Design |  |  |
|  | Group Size 5 | 2,224,260 |
|  | Group Size 10 | 2,402,440 |
|  | Group Size 20 | 2,585,830 |
| Two-Phase Group Testing Design |  |  |
|  | Group Size 5 | 2,636,000 |
|  | Group Size 10 | 1,853,500 |
|  | Group Size 20 | 1,100,750 |

Table S4. Expected Number of Tests by Design with Example Data for Cutoff of 1.15.

The number of tests used for the case control design, the standard group testing design, and the expected number of tests for the two-phase group testing design with the cutoff adjusted to 1.15
